# Health services utilization, out‐of‐pocket expenditure, and underinsurance among insured non‐elderly cancer survivors in the United States, 2011–2015

**DOI:** 10.1002/cam4.4103

**Published:** 2021-07-30

**Authors:** Mohammad A. Karim, Amit G. Singal, Robert L. Ohsfeldt, Michael A. Morrisey, Hye‐Chung Kum

**Affiliations:** ^1^ Population Informatics Laboratory, Department of Health Policy and Management, School of Public Health Texas A&M University College Station TX USA; ^2^ Division of Digestive and Liver Diseases University of Texas Southwestern Medical Center Dallas TX USA; ^3^ Present address: Cancer Prevention Research Training Program Department of Epidemiology, Division of Cancer Prevention and Population Sciences The University of Texas MD Anderson Cancer Center Houston TX USA

**Keywords:** cancer, health services, insurance, out‐of‐pocket expenditure

## Abstract

**Background:**

High out‐of‐pocket (OOP) expenditure and inadequate insurance coverage may adversely affect cancer survivors. We aimed to characterize the extent and correlates of healthcare utilization, OOP expenditures, and underinsurance among insured cancer survivors.

**Methods:**

We used 2011–2015 Medical Expenditure Panel Survey data to identify a nationally representative sample of insured non‐elderly adult (age 18–64 years) cancer survivors. We used negative binomial, two‐part (logistic and Generalized Linear Model with log link and gamma distribution), and logistic regression models to quantify healthcare utilization, OOP expenditures, and underinsurance, respectively, and identified sociodemographic correlates for each outcome.

**Results:**

We identified 2738 insured non‐elderly cancer survivors. Adjusted average utilization of ambulatory, non‐ambulatory, prescription medication, and dental services was 14.4, 0.51, 24.9, and 1.4 events per person per year, respectively. Higher ambulatory and dental services utilization were observed in older adults, females, non‐Hispanic Whites, survivors with a college degree and high income, compared to their counterparts. Nearly all (97.7%) survivors had some OOP expenditures, with a mean adjusted OOP expenditure of $1552 per person per year. Adjusted mean OOP expenditures for ambulatory, non‐ambulatory, prescription medication, dental, and other health services were $653, $161, $428, $194, and $83, respectively. Sociodemographic variations in service‐specific OOP expenditures were generally consistent with respective utilization patterns. Overall, 8.8% of the survivors were underinsured.

**Conclusion:**

Many insured non‐elderly cancer survivors allocate a substantial portion of their OOP expenditure for healthcare‐related services and experience financial vulnerability, resulting in nearly 8.8% of the survivors being underinsured. Utilization of healthcare services varies across sociodemographic groups.

## Graphical abstract

This paper estimates services utilization, out‐of‐pocket (OOP) expenditure, and underinsurance among insured non‐elderly adult cancer survivors. We provide novel evidence on sociodemographic variations in health services utilization and service‐specific OOP expenditure among cancer survivors using Medical Expenditure Panel Survey data.

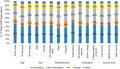

## 1 INTRODUCTION

Despite the decreasing mortality in the last 25 years, cancer remains a deadly disease with more than 600,000 estimated deaths in the United States in 2020.^1^ Cancer is also associated with significant morbidity with an adverse impact on quality of life among survivors.^2^, ^3^ Besides its mortality and morbidity impact, the adverse financial impact of cancer on survivors, oftentimes called “financial toxicity,” has become a matter of grave concern among survivors, providers, and policy makers.^4^, ^5^, ^6^ With the rising cost of healthcare, fueled by the introduction of new technologies and medications, survivors are prone to high out‐of‐pocket (OOP) costs.^7^, ^8^, ^9^ The high cost of services may negatively impact the care received and overall well‐being of survivors. Studies have reported non‐compliance, forgone medication purchases, and high level of hardship experienced by cancer survivors related to financial toxicity.^10^, ^11^

Although several studies have examined uninsured cancer survivors, those with insurance coverage are not immune to financial toxicity.^12^, ^13^ Due to variability in OOP maximum provisions between health plans, OOP expenditure burden on cancer survivors can become substantial.^14^, ^15^ Moreover, the OOP burden may vary depending on cancer management strategies.^16^, ^17^ In addition to cancer‐specific costs, unrelated medical care for comorbid conditions may exacerbate financial burden.^18^

The American Society of Clinical Oncology Guidance on Cost of Cancer Care identified patient–provider discussions about costs as a key component of high quality care.^19^ Stakeholders involved in different points of cancer care spectrum have suggested price transparency and awareness, medication price related and payment model‐related policy revisions, and enhanced patient engagement as potential steps to contain the financial toxicity of cancer.^6^, ^20^ The multifaceted aspect of financial toxicity of cancer makes it a challenging problem that warrants collaborative and well‐informed actions from all stakeholders.

Prior studies have reported high financial burden of medical care among cancer survivors, although there are limited data examining how service‐specific utilization and OOP expenditure varies among subgroups.^14^, ^15^, ^21^, ^22^, ^23^, ^24^, ^25^ The purpose of our current study was to investigate sociodemographic variations in healthcare utilization, OOP expenditures, and underinsurance among a large nationally representative sample of insured cancer survivors in the United States.

## 2 METHODS

### 2.1 Data source and study sample

We used the Full Year Consolidated (FYC) files and the Medical Conditions (MC) files of the Household Component of Medical Expenditure Panel Survey (MEPS) for the years 2011–2015. We identified non‐elderly adult (age 18–64 years) cancer survivors using Clinical Classifications Software codes 21–45 from the MC files and linked the information to FYC files. We identified 2738 non‐elderly cancer survivors for whom cancer was reported as a current condition and complete data were available. A medical condition which a respondent was experiencing or had an event linked to during the survey year is defined as a “current condition” in MEPS.^26^ Our purpose was to examine the insured cancer survivors; thus, only the survivors with full year insurance coverage were included in this study. Expenditure and utilization data were obtained from MEPS FYC files. Services‐specific utilization and expenditure data are primarily self‐reported in the household survey with a subset of the data confirmed with providers through the Medical Provider Component of MEPS.^27^ Although expenditures may be underreported in MEPS, utilization data for services such as prescription medication purchase and non‐ambulatory visits are demonstrated to be fairly accurate.^28^, ^29^, ^30^ MEPS employs a multistage survey on a nationally representative sample of civilian non‐institutionalized population in the United States that oversamples minority racial/ethnic groups (Blacks, Hispanics, and Asians); so, to obtain national level estimates it is necessary to account for the survey design.^27^, ^31^ We conducted all our analyses using the survey‐specific commands in Stata software (StataCorp), incorporating MEPS reported survey weight, strata, and primary sampling unit variables in our statistical models.^32^, ^33^

### 2.2 Measures

#### 2.2.1 Health services utilization

Health services utilization was quantified separately for ambulatory, non‐ambulatory, prescription medication, and dental services. Ambulatory care utilization was the total number of office based and outpatient visits, non‐ambulatory utilization was the total number of inpatient discharges and emergency room (ER) visits, prescription medication utilization was the total number of prescription medication purchase events (including refills), and dental care utilization was the total number of dental visits per person over 1‐year period.^27^, ^34^

#### 2.2.2 Total and service‐specific OOP expenditure

Total OOP expenditure was the sum of a cancer survivor's expenditure for all health services utilized over 1‐year period.^35^ Separate service‐specific OOPs were also estimated in our analysis for ambulatory (office based + outpatient), non‐ambulatory (inpatient + ER), prescription medication (initial purchase + refills), dental services, and other health services (home health + vision + device + others). Cancer‐related versus unrelated services were not differentiated in either utilization or OOP estimates.

#### 2.2.3 Underinsurance

Following previous studies, underinsurance was defined using an indicator variable based on the ratio of total OOP and family income (FI). Specifically, it was defined as total OOP ≥5% of FI for FI < 200% federal poverty level (FPL) or ≥10% of FI for FI ≥ 200% FPL, for the individuals having full year insurance coverage.^33^, ^36^ This concept of underinsurance has the advantage of quantifying financial inadequacy based on varying OOP to FI ratio,^33^, ^36^, ^37^, ^38^ versus the commonly reported threshold of OOP ≥20% of FI among all survivors.^15^, ^24^, ^25^ However, we performed two sensitivity analyses using two different underinsurance thresholds: one using a fixed threshold at OOP ≥20% of FI for all income levels and another using a sliding threshold of OOP ≥5%, ≥10%, ≥15%, and ≥20% of FI for FI < 125%, 125% to <200%, 200% to <400%, and >400% of FPL, respectively.

#### 2.2.4 Covariates

Age (18–49, 50–59, and 60–64 years), sex (male and female), race/ethnicity (non‐Hispanic White, Black, Hispanic, and Asian/others), marital status (not married and married), income level (low [<200% of FPL], middle [200% to <400% of FPL], and high income [≥400% of FPL]), education (high school education/diploma, some college, college degree, or above), insurance status (private––managed care, private––non‐managed care, Medicaid, and Medicare/dual‐eligible), number of MEPS priority conditions (none, one, two, three, or more), self‐reported health status (poor/fair, good, and very good/excellent), and census region (northeast, midwest, south, and west) were included as covariates in each estimation model. Number of MEPS priority conditions (i.e., comorbid conditions investigated in MEPS due to their prevalence, expense, or policy significance),^26^ excluding cancer and attention deficit hyperactivity disorder, was a categorical variable based on the actual number of conditions. (Table 1).

**TABLE 1:** Sample characteristics: insured non‐elderly cancer survivors from the Medical Expenditure Panel Survey, 2011–2015 (*N* = 2738)

Variables	Categories	Unweighted *n*	Weighted %
Age (years)	18–49	929	31.6
	50–59	1085	40.9
	60–64	724	27.6
Sex	Male	988	39.5
	Female	1750	60.5
Race/ethnicity	Non‐Hispanic White	1745	81.8
	Black	415	7.1
	Hispanic	398	7.1
	Asian/others	180	4.0
Marital status	Not married	1081	33.2
	Married	1657	66.8
Education	HS education/diploma	989	29.5
	Some college	827	30.7
	College degree or above	922	39.8
Income level^a^	Low income	795	20.1
	Middle income	709	24.0
	High income	1234	56.0
Insurance status	Private MC	604	22.3
	Private non‐MC	1506	62.5
	Medicaid	356	7.7
	Medicare/dual‐eligible	272	7.5
Number of MEPS priority conditions	None	432	14.6
	One	515	19.9
	Two	523	19.9
	Three or more	1268	45.6
Census region	Northeast	549	20.2
	Midwest	578	22.3
	South	928	34.9
	West	683	22.6
Health status	Poor or fair	756	22.7
	Good	831	28.4
	Very good or excellent	1151	48.9

Note

Survey weighted descriptive statistics based on the analysis of MEPS data (2011–2015).

Abbreviations: FI, family income; FPL, federal poverty level; HS, high school; MC, managed care; MEPS, Medical Expenditure Panel Survey.

^a^ Low income represents FI < 200% of FPL, middle income represents FI 200% to <400% of FPL, and high income represents FI ≥ 400% of FPL.

### 2.3 Statistical analysis

We evaluated three outcomes: health services utilization, OOP expenditure, and underinsurance. Health services utilization was estimated using negative binomial model for each service type where the total number of events per person per year was the dependent variable. Total and service‐specific OOPs were estimated using two‐part regression models (logistic and Generalized Linear Model (GLM) with log link and gamma distribution).^35^ GLM‐only sensitivity analyses were performed to test the effect of estimation method variation. The prevalence of underinsurance was estimated using a multivariable logistic regression model. Average adjusted prediction yielded the point estimates and average marginal effect yielded variations and respective statistical significance across each sociodemographic variable.^35^, ^39^, ^40^ We conducted subgroup analyses by sociodemographic factors including age, sex, race/ethnicity, education, income, and insurance status. Income and expenditure dollar values were inflated to 2018 US dollars using the consumer price index and values were rounded.^41^ Statistical significance was defined at a 5% level. Data management was performed in SAS 9.4 (SAS Institute, Inc.) and all analyses were performed in Stata 13.1 (StataCorp).

## 3 RESULTS

### 3.1 Sample characteristics

Our analytic cohort consisted of 2738 non‐elderly cancer survivors. The overall weighted sample was majority White (81.8%), aged 50–59 years (40.9%), female dominant (60.5%), and married (66.8%). Majority of the sample had a college degree or above (39.8%), high income (56%), and private non‐managed care insurance coverage (62.5%). Although 45.6% reported three or more comorbid conditions, nearly half of the sample (48.9%) reported very good or excellent health status (Table 1).

### 3.2 Estimates and correlates of health services utilization

**FIGURE 1:**
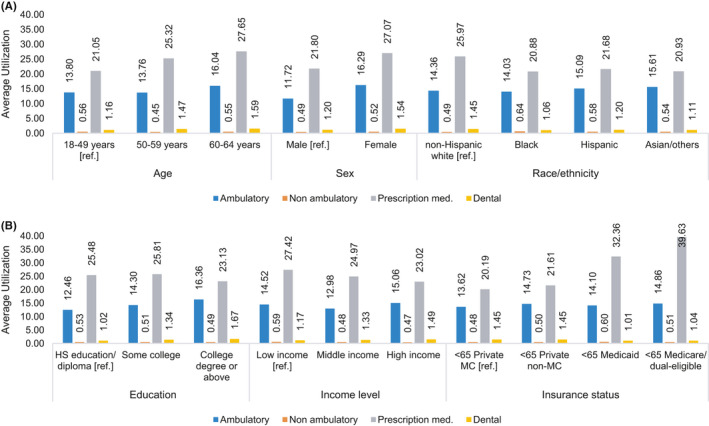
Average services utilization per person per year by (A) demographic characteristics, (B) socioeconomic characteristics, among insured non‐elderly cancer survivors, 2011–2015. Estimates represent average adjusted prediction (AAP) from negative binomial model for each service type. Estimation models were adjusted for age, sex, race/ethnicity, marital status, income level, education, census region, insurance status, number of Medical Expenditure Panel Survey (MEPS) priority conditions, and self‐reported health status. Low income represents family income (FI) <200% of federal poverty level (FPL), middle income represents FI 200% to <400% of FPL, and high income represents FI ≥400% of FPL

Adjusted mean utilization of ambulatory, non‐ambulatory, prescription medication, and dental services for the full cohort was 14.4, 0.51, 24.9, and 1.4 events per person per year, respectively. Survivors aged 60–64 years and females had significantly higher ambulatory, prescription medication, and dental services utilization compared to those aged 18–49 years and males, respectively (Figure 1; Table S1). Utilization of dental care was substantially lower among racial/ethnic minorities, survivors with high school education and low income compared to survivors with non‐Hispanic White race/ethnicity, a college degree and high income, respectively (Figure 1; Table S1). Non‐ambulatory services utilization was consistent across subgroups.

### 3.3 Estimates and correlates of OOP expenditures

**FIGURE 2:**
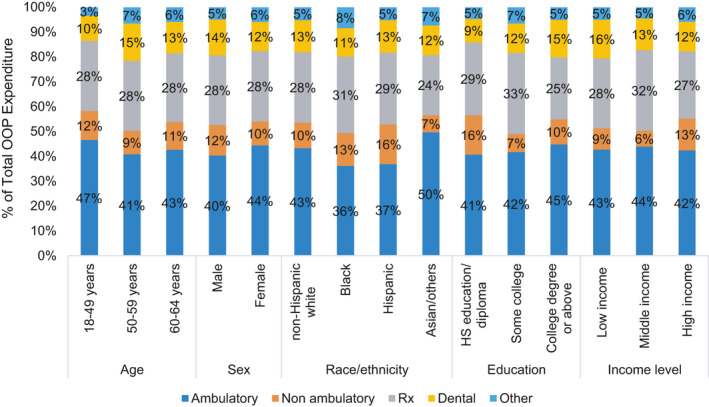
Percent contribution of service‐specific out‐of‐pocket (OOP) expenditure to total OOP expenditure by sociodemographic characteristics, non‐elderly cancer survivors, 2011–2015. Low income represents family income (FI) <200% of federal poverty level (FPL), middle income represents FI 200% to <400% of FPL, and high income represents FI ≥400% of FPL

Nearly all (97.7%) survivors had some OOP expenditure, with adjusted mean OOP per person per year of $1552. Adjusted mean OOP expenditures for ambulatory, non‐ambulatory, prescription medication, dental, and other health services for the full cohort were $653, $161, $428, $194, and $83, respectively. Survivors spent the highest proportion of their total OOP on ambulatory services and the second highest on prescription medications (Figure 2).

OOP expenditure pattern for different health services varied by demographic subgroups with survivors aged 50–59 years and 60–64 years spending more on dental and other health services; females spending more on ambulatory, prescription medication, and other health services; and Black survivors spending less on ambulatory, prescription medication, and dental services compared to their respective counterparts (Table 2).

**TABLE 2:** Adjusted out‐of‐pocket expenditure by sociodemographic characteristics, insured non‐elderly cancer survivors, 2011–2015

	Ambulatory		Non‐ambulatory		Prescription medications		Dental		Others	
	Mean OOP^a^	*p* ^b^	Mean OOP^a^	*p* ^b^	Mean OOP^a^	*p* ^b^	Mean OOP^a^	*p* ^b^	Mean OOP^a^	*p* ^b^
Age (years)										
18–49 [ref.]	632		157		384		136		47	
50–59	590	0.537	136	0.546	407	0.682	217	0.038	95	<0.001
60–64	763	0.092	201	0.425	493	0.082	227	0.027	105	0.001
Sex										
Male [ref.]	544		166		379		195		66	
Female	727	<0.001	158	0.845	464	0.047	194	0.972	96	0.003
Race/ethnicity										
Non‐Hispanic White [ref.]	691		164		450		205		84	
Black	340	<0.001	125	0.463	289	<0.001	108	0.021	79	0.816
Hispanic	485	0.009	210	0.549	379	0.264	171	0.379	70	0.346
Asian/others	602	0.547	84	0.068	293	0.007	144	0.189	89	0.885
Education										
HS education/diploma [ref.]	532		207		384		124		61	
Some college	591	0.322	103	0.120	462	0.195	167	0.134	93	0.028
College degree or above	784	<0.001	178	0.622	436	0.288	263	<0.001	90	0.045
Income level^c^										
Low income [ref.]	538		107		354		196		64	
Middle income	603	0.406	87	0.565	444	0.155	174	0.619	63	0.962
High income	710	0.036	214	0.099	451	0.105	202	0.890	97	0.022
Insurance status										
Private MC [ref.]	625		128		392		240		98	
Private non‐MC	732	0.062	197	0.106	446	0.204	197	0.269	79	0.206
Medicaid	329	0.002	17	0.003	129	<0.001	41	<0.001	37	0.001
Medicare/dual‐eligible	367	0.003	119	0.892	634	0.040	168	0.478	114	0.678

Abbreviations: AAP, average adjusted prediction; AME, average marginal effect; FI, family income; FPL, federal poverty level; HS, high school; MC, managed care; MEPS, Medical Expenditure Panel Survey; OOP, out‐of‐pocket.

^a^ AAP from a two‐part model (first part: logistic, second part: Generalized Linear Model (GLM) with log link and gamma distribution). Estimation model was adjusted for age, sex, race/ethnicity, marital status, income level, education, census region, insurance status, number of MEPS priority conditions, and self‐reported health status.

^b^ *p*‐Values represent statistical significance of AME contrasting the AAP of each category to the AAP of the reference category (the first row) for each variable.

^c^ Low income represents FI <200% of FPL, middle income represents FI 200% to <400% of FPL, and high income represents FI ≥400% of FPL.

Cancer survivors with high socioeconomic status generally incurred higher OOP expenditure, with survivors having a college degree and high income spending more on ambulatory services compared to those with high school education and low income, respectively (Table 2). Additionally, variation in insurance status was associated with variation in service‐specific OOP expenditure with survivors covered by Medicaid incurring significantly lower OOP for all health services compared to those covered by private managed care plans (Table 2).

### 3.4 Prevalence and correlates of underinsurance

Overall, 8.8% of non‐elderly cancer survivors with insurance were identified as underinsured in the adjusted analysis. In subgroup analyses, underinsurance was more common in older adults aged 60–64 years, non‐Hispanic Whites, and survivors with a college degree compared to their respective counterparts (Table 3). Underinsurance was less common among middle‐ and high‐income survivors compared to those with low income. Survivors with Medicaid and Medicare/dual‐enrollment were less likely to be underinsured compared to survivors with private managed care plans (Table 3).

**TABLE 3:** Adjusted probability of underinsurance by sociodemographic characteristics, insured non‐elderly cancer survivors, 2011–2015

	Probability of underinsurance (%)^a^	*p* ^b^
Age (years)		
18–49 [ref.]	6.21	
50–59	8.17	0.114
60–64	13.02	<0.001
Sex		
Male [ref.]	9.09	
Female	8.68	0.696
Race/ethnicity		
Non‐Hispanic White [ref.]	9.8	
Black	4.57	<0.001
Hispanic	7.79	0.117
Asian/others	6.21	0.047
Education		
HS education/diploma [ref.]	7.46	
Some college	9.37	0.142
College degree or above	11.07	0.021
Income level^c^		
Low income [ref.]	45.36	
Middle income	5.77	<0.001
High income	0.7	<0.001
Insurance status		
Private MC [ref.]	11.99	
Private non‐MC	11.86	0.941
Medicaid	3.65	<0.001
Medicare/dual‐eligible	6.54	0.008

Abbreviations: AAP, average adjusted prediction; AME, average marginal effect; FI, family income; FPL, federal poverty level; HS, high school; MC, managed care; MEPS, Medical Expenditure Panel Survey.

^a^ AAP from a logistic model. Estimation model was adjusted for age, sex, race/ethnicity, marital status, income level, education, census region, insurance status, number of MEPS priority conditions, and self‐reported health status.

^b^ *p*‐values represent statistical significance of AME contrasting the AAP of each category to the AAP of the reference category (the first row) for each variable.

^c^ Low income represents FI < 200% of FPL, middle income represents FI 200% to <400% of FPL, and high income represents FI ≥ 400% of FPL.

### 3.5 Sensitivity analysis

We performed several sensitivity analyses to check the robustness of our estimates. A GLM‐only model for total OOP instead of a two‐part model of the base case found very similar estimates, although service‐specific OOP estimates had greater variations. The mean total OOP estimates from two‐part and GLM‐only models were: $1552 and $1559, respectively.

Adjusted probability of underinsurance was 2.9% for a fixed threshold of OOP ≥20% of FI; and was 6.4% for a sliding threshold of OOP ≥5%, ≥10%, ≥15%, and ≥20% of FI for FI < 125%, 125% to <200%, 200% to <400%, and >400% of FPL, respectively.

## 4 DISCUSSION

Our study highlights that many insured non‐elderly cancer survivors require high services utilization, resulting in high OOP expenditures. Of particular concern, we found nearly 8.8% of the non‐elderly cancer survivors were underinsured.

We observed some consistent findings in subgroup analyses that are worth highlighting. First, older adults nearing Medicare eligibility (i.e., age 60–64 years) had significantly higher services utilization, higher OOP expenditures, and increased underinsurance than younger individuals. This is likely related to increased comorbidity, suggesting a need for specific insurance reform for this age group.^42^ Second, we observed higher services utilization and OOP expenditure among female survivors. This result, in conjunction with a previous finding of females being 27% more likely to experience cost‐related medication non‐adherence, indicates a heightened risk of non‐adherence among female cancer survivors.^43^ Our finding of higher OOP among female survivors are consistent with previous report of higher overall healthcare expenditure incurred for females (vs. males) in general population.^44^ Among female cancer survivors of childbearing age, interest in fertility preservation interventions has been reported, which may play a role in higher OOP expenditure.^45^ Among older non‐elderly females aged between 50 and 64 years, postmenopausal healthcare, cardiovascular diseases, and osteoporosis have been identified as potential drivers of non‐cancer OOP expenditures,^44^ which might be responsible for female cancer survivors’ higher health‐related OOP expenses compared to males. Third, we observed consistently lower utilization of several health services (i.e., ambulatory and dental care), lower OOP costs, and underinsurance among survivors of Black race/ethnicity and low educational attainment. Despite lower OOP and underinsurance estimates, lower utilization among these groups is concerning. A preponderance of data shows increased disease burden and worse clinical outcomes in socially disadvantaged cancer population.^46^, ^47^, ^48^, ^49^ The lower OOP and underinsurance pattern among these groups may be driven by increased barriers and decreased access to healthcare, rather than lower healthcare needs.

Black cancer survivors are more likely to receive care from limited resource settings, reducing their access to health services.^46^ A 2017 study demonstrated that insured individuals among the most socially disadvantaged cancer survivors are less likely to receive cancer‐directed surgery compared to the least socially disadvantaged survivors.^50^ Additionally, lower health literacy may adversely affect the healthcare utilization by minorities.^51^ It is well established that historic discriminations, limited access to education, racism, and cultural insensitivity have hampered the ability of African American population to adequately acquire and interpret health information, affecting their ability to utilize needed healthcare.^51^ Moreover, Black and Hispanic cancer survivors are more likely to forego necessary healthcare due to cost burden,^52^ which may result in missed underinsurance in some survivors. Thus, lower receipt of services due to access and health literacy barriers and underutilization of services due to cost are the likely reasons behind the apparent lower OOP expenditure and underinsurance observed among the insured socially disadvantaged groups in our study. These access and utilization issues warrant policy attention while formulating financial toxicity‐mitigating strategies.

Prescription medication costs have come under increased scrutiny given the upward trend in cancer therapy pricing.^4^, ^53^, ^54^ Industry practices, such as drug companies increasing the prices of anticancer medications after obtaining desired market share, may exacerbate financial toxicity of prescription medications.^55^ In addition to high resultant OOP costs, high drug costs may also result in medication non‐adherence as a means to control OOP.^10^, ^56^ These issues are not only important for clinicians to consider when selecting between medication choices but also highlight a need for policy changes to curb rising cancer medication costs; particularly to ease survivor OOP burden which accounted for 24% or higher percentage of total OOP for all sociodemographic groups in our study (Figure 2). Specially concerning was our finding that despite allocating similar or higher proportion of their total spending on prescription medications, all minority groups had substantially lower prescription medication utilization compared to non‐Hispanic Whites (Figures 1 and 2). Additionally, we found non‐cancer‐related services, like dental care, constituted a substantial portion of financial burden for cancer survivors, while sociodemographic variations in utilization persisted; survivors of non‐White race/ethnicity, with high school education/diploma, and low income utilized less dental care. This might be an indication of financial toxicity of cancer adversely affecting disadvantaged survivors’ utilization of non‐essential but beneficial health services. These findings of lower utilization are consistent with previous reports of Black and Hispanic cancer survivors’ higher likelihood of foregoing prescription medication and dental services due to cost burden.^52^

The Affordable Care Act (ACA) made cancer screening more affordable and expanded Medicaid, which resulted in increased preventive services utilization, early‐stage cancer detection, and increased services utilization.^57^, ^58^, ^59^ Our study shows that non‐elderly Medicaid covered survivors incurred significantly less OOP expenditure for all health service types compared to survivors covered by private managed care plans; however, we were unable to identify survivors with exchange plans. A previous population level study demonstrated that marketplace plans cause higher OOP costs among near‐poor adults compared to Medicaid.^60^ Keeping this in consideration, future studies should investigate how the OOP and utilization of cancer survivors with marketplace plans compare to those with other insurance plans. Following ACA’s success in increasing the number of covered individuals,^61^ improving the quality of insurance plans should also be part of healthcare reform considerations. Although ACA instituted OOP expenditure limits starting 2014, the burden on low‐income individuals remains substantial. In 2020, the $8150 OOP limit on marketplace plans for one‐person household was about 48% and 32% of income for a one‐person household earning at 133% and 200% of FPL, respectively.^62^, ^63^ Among our overall sample, the estimated prevalence of underinsurance was 8.8%, which is lower than the 21% population level underinsurance estimate reported in a 2020 Commonwealth Fund publication.^64^ Different definitions of underinsurance may be a possible explanation behind this difference in estimates: while the Commonwealth Fund considered individuals having deductible 5% or more of their household income as underinsured, we could not incorporate deductibles in our underinsurance indicator due to lack of deductible related information in MEPS.^64^ However, we found that the prevalence of underinsurance among low‐income (FI < 200% of FPL) survivors was 45.36%, which is consistent with the high burden estimates reported by Bernard et al., and is higher than the estimates reported by Guy et al. among similar subgroups of cancer survivors.^15^, ^23^ Our study demonstrates that inadequate insurance protection against financial toxicity and utilization variations among non‐elderly cancer survivors is prevalent, which highlights the need for increased high‐quality coverage.

We would like to note a few limitations of our study. First, MEPS expenditure and utilization data are patient‐reported, with a sub‐sample cross‐checked with the providers, so there is potential recall bias.^65^ Second, underinsurance estimates may have been underestimated because insurance premium was not included in OOP expenditure and high‐deductible cases could not be identified; although prior MEPS‐based studies reported use of similar underinsurance measures.^66^ Third, we were unable to perform subgroup analyses by cancer type due to sample size limitations. Fourth, we identified cancer survivors using MEPS definition of “current condition,” which cannot be interpreted as active treatment: survivors who had a healthcare event related to a specific health condition in the survey year or who reported to be experiencing a specific health condition during the survey, both were identified to have a “current condition” in MEPS.^26^ Finally, tumor stage, time since diagnosis, or other clinically relevant variables could not be included in our analyses because MEPS does not provide these data.

In this study, we found many insured non‐elderly cancer survivors have high services utilization and OOP expenditures resulting in nearly 8.8% being underinsured. Lower health services utilization by the underserved cancer survivors indicates that the real extent of the financial hardship may be much worse. This highlights the need to take healthcare access issues into consideration while formulating policies to mitigate financial toxicity. Our study underscores the continued need for further policy changes in health insurance coverage and healthcare access, including among cancer survivors, in the United States.

## ETHICAL APPROVAL STATEMENT

Ethical approval for this study was sought from the Texas A&M University Institutional Review Board and upon initial review they determined the study to be “not research involving human subjects as defined by DHHS and FDA regulations” and “further IRB review and approval by this organization is not required because this is not human research”. Determination date: 01/15/2020. Reference number: 104335.

## CONFLICT OF INTEREST

The authors disclose no conflict of interest.

## Supporting information

Table S1

## Data Availability

The data that support the findings of this study were derived from the following resources available in the public domain: https://meps.ahrq.gov/data_stats/download_data_files.jsp
