# Supplementary material for: Health services utilization, out‐of‐pocket expenditure, and underinsurance among insured non‐elderly cancer survivors in the United States, 2011–2015
Source: Cancer Med. 2021 Jul 30;10(16):5513–23. doi: 10.1002/cam4.4103 (PMC8366084; doi:10.1002/cam4.4103)
Supplement: Supplementary file 1 — Table S1 [file CAM4-10-5513-s001.docx]

# Table S1. Adjusted health services utilization by sociodemographic characteristics, insured non-elderly cancer survivors, 2011–2015

|  |  | **Ambulatory** | | **Non-ambulatory** | | **Prescription medications** | | **Dental** | |
| --- | --- | --- | --- | --- | --- | --- | --- | --- | --- |
|  |  | **Mean no. of events ^†^** | **P ^‡^** | **Mean no. of events ^†^** | **P ^‡^** | **Mean no. of events ^†^** | **P ^‡^** | **Mean no. of events ^†^** | **P ^‡^** |
| **Age** | 18–49 years [ref.] | 13.80 |  | 0.56 |  | 21.05 |  | 1.16 |  |
|  | 50–59 years | 13.76 | 0.955 | 0.45 | 0.078 | 25.32 | 0.020 | 1.47 | 0.001 |
|  | 60–64 years | 16.04 | 0.014 | 0.55 | 0.867 | 27.65 | 0.001 | 1.59 | 0.001 |
| **Sex** | Male [ref.] | 11.72 |  | 0.49 |  | 21.80 |  | 1.20 |  |
|  | Female | 16.29 | 0.000 | 0.52 | 0.588 | 27.07 | 0.002 | 1.54 | 0.001 |
| **Race/ethnicity** | Non-Hispanic White [ref.] | 14.36 |  | 0.49 |  | 25.97 |  | 1.45 |  |
|  | Black | 14.03 | 0.751 | 0.64 | 0.089 | 20.88 | 0.015 | 1.06 | 0.019 |
|  | Hispanic | 15.09 | 0.564 | 0.58 | 0.301 | 21.68 | 0.052 | 1.20 | 0.121 |
|  | Asian/others | 15.61 | 0.454 | 0.54 | 0.569 | 20.93 | 0.056 | 1.11 | 0.040 |
| **Education** | HS education/diploma [ref.] | 12.46 |  | 0.53 |  | 25.48 |  | 1.02 |  |
|  | Some college | 14.30 | 0.023 | 0.51 | 0.797 | 25.81 | 0.874 | 1.34 | 0.010 |
|  | College degree or above | 16.36 | 0.000 | 0.49 | 0.586 | 23.13 | 0.184 | 1.67 | 0.000 |
| **Income level ^§^** | Low income [ref.] | 14.52 |  | 0.59 |  | 27.42 |  | 1.17 |  |
|  | Middle income | 12.98 | 0.169 | 0.48 | 0.187 | 24.97 | 0.344 | 1.33 | 0.222 |
|  | High income | 15.06 | 0.668 | 0.47 | 0.123 | 23.02 | 0.138 | 1.49 | 0.024 |
| **Insurance status** | Private MC [ref.] | 13.62 |  | 0.48 |  | 20.19 |  | 1.45 |  |
|  | Private non-MC | 14.73 | 0.230 | 0.50 | 0.731 | 21.61 | 0.339 | 1.45 | 0.992 |
|  | Medicaid | 14.10 | 0.754 | 0.60 | 0.262 | 32.36 | 0.000 | 1.01 | 0.027 |
|  | Medicare/dual-eligible | 14.86 | 0.366 | 0.51 | 0.762 | 39.63 | 0.000 | 1.04 | 0.057 |

Abbreviations: HS, High school; MC, Managed care.

Note: **^†^** Average adjusted prediction (AAP) from a negative binomial model. Estimation model was adjusted for age, sex, race/ethnicity, marital status, income level, education, census region, insurance status, number of MEPS priority conditions, and self-reported health status.

**^‡^** *p*-values represent statistical significance of average marginal effect (AME) contrasting the AAP of each category to the AAP of the reference category (the first row) for each variable.

**^§^** Low income represents family income (FI) <200% of federal poverty level (FPL), middle income represents FI 200% to <400% of FPL, and high income represents FI ≥400% of FPL.

# **APPENDIX**

| Supplemental Table 1. Adjusted health services utilization by sociodemographic characteristics, insured non-elderly cancer survivors, 2011–2015 | | | | | | | | | |
| --- | --- | --- | --- | --- | --- | --- | --- | --- | --- |
|  |  | **Ambulatory** | | **Non-ambulatory** | | **Prescription medications** | | **Dental** | |
|  |  | **Mean no. of events ^†^** | **P ^‡^** | **Mean no. of events ^†^** | **P ^‡^** | **Mean no. of events ^†^** | **P ^‡^** | **Mean no. of events ^†^** | **P ^‡^** |
| **Age** | 18–49 years [ref.] | 13.80 |  | 0.56 |  | 21.05 |  | 1.16 |  |
|  | 50–59 years | 13.76 | 0.955 | 0.45 | 0.078 | 25.32 | 0.020 | 1.47 | 0.001 |
|  | 60–64 years | 16.04 | 0.014 | 0.55 | 0.867 | 27.65 | 0.001 | 1.59 | 0.001 |
| **Sex** | Male [ref.] | 11.72 |  | 0.49 |  | 21.80 |  | 1.20 |  |
|  | Female | 16.29 | 0.000 | 0.52 | 0.588 | 27.07 | 0.002 | 1.54 | 0.001 |
| **Race/ethnicity** | Non-Hispanic White [ref.] | 14.36 |  | 0.49 |  | 25.97 |  | 1.45 |  |
|  | Black | 14.03 | 0.751 | 0.64 | 0.089 | 20.88 | 0.015 | 1.06 | 0.019 |
|  | Hispanic | 15.09 | 0.564 | 0.58 | 0.301 | 21.68 | 0.052 | 1.20 | 0.121 |
|  | Asian/others | 15.61 | 0.454 | 0.54 | 0.569 | 20.93 | 0.056 | 1.11 | 0.040 |
| **Education** | HS education/diploma [ref.] | 12.46 |  | 0.53 |  | 25.48 |  | 1.02 |  |
|  | Some college | 14.30 | 0.023 | 0.51 | 0.797 | 25.81 | 0.874 | 1.34 | 0.010 |
|  | College degree or above | 16.36 | 0.000 | 0.49 | 0.586 | 23.13 | 0.184 | 1.67 | 0.000 |
| **Income level ^§^** | Low income [ref.] | 14.52 |  | 0.59 |  | 27.42 |  | 1.17 |  |
|  | Middle income | 12.98 | 0.169 | 0.48 | 0.187 | 24.97 | 0.344 | 1.33 | 0.222 |
|  | High income | 15.06 | 0.668 | 0.47 | 0.123 | 23.02 | 0.138 | 1.49 | 0.024 |
| **Insurance status** | Private MC [ref.] | 13.62 |  | 0.48 |  | 20.19 |  | 1.45 |  |
|  | Private non-MC | 14.73 | 0.230 | 0.50 | 0.731 | 21.61 | 0.339 | 1.45 | 0.992 |
|  | Medicaid | 14.10 | 0.754 | 0.60 | 0.262 | 32.36 | 0.000 | 1.01 | 0.027 |
|  | Medicare/dual-eligible | 14.86 | 0.366 | 0.51 | 0.762 | 39.63 | 0.000 | 1.04 | 0.057 |
|  |  |  |  |  |  |  |  |  |  |
| Abbreviations: HS, High school; MC, Managed care.  Note: **^†^** Average adjusted prediction (AAP) from a negative binomial model. Estimation model was adjusted for age, sex, race/ethnicity, marital status, income level, education, census region, insurance status, number of MEPS priority conditions, and self-reported health status.  **^‡^** *p*-values represent statistical significance of average marginal effect (AME) contrasting the AAP of each category to the AAP of the reference category (the first row) for each variable.  **^§^** Low income represents family income (FI) <200% of federal poverty level (FPL), middle income represents FI 200% to <400% of FPL, and high income represents FI ≥400% of FPL. | | | | | | | | | |
